# Supplementary material for: Proportions and determinants of successful surgical repair of obstetric fistula in low- and middle-income countries: A systematic review and meta-analysis
Source: PLoS One. 2024 May 9;19(5):e0303020. doi: 10.1371/journal.pone.0303020 (PMC11081269; doi:10.1371/journal.pone.0303020)
Supplement: S1 Table — (DOCX) [file pone.0303020.s003.docx]

**Supplementary file III: A table showing the risk of bias assessment of the included studies.**

| **Author** | **Publication Year** | **Design** | **JBI Critical appraisal tool** | **Score in number** | **Score in percentage** | **Risk of bias category** |
| --- | --- | --- | --- | --- | --- | --- |
| Gezimu W, et al. (1) | 2023 | Retrospective cross-sectional | Analytical cross-sectional | 7/8 | 87.5 | Low |
| Traore TM, et al. (2) | 2023 | Retrospective cross-sectional | Prevalence studies | 7/8 | 87.5 | Low |
| Kumsa MH, et al. (3) | 2023 | Retrospective cross-sectional | Analytical cross-sectional | 7/8 | 87.5 | Low |
| Niragira J, Wang TX (4) | 2023 | Cross-sectional | Analytical cross-sectional | 7/8 | 87.5 | Low |
| Patel TB, et al (5) | 2023 | Retrospective cross-sectional | Analytical cross-sectional | 6/8 | 75 | Low |
| Ambese TY, et al. (6) | 2022 | Retrospective cohort | Cohort studies | 8/11 | 72.7 | Low |
| Tadesse et al. (7) | 2022 | Cross-sectional | Analytical cross-sectional | 8/8 | 100 | Low |
| Asif M, et al (8) | 2022 | Cross-sectional | Prevalence studies | 7/9 | 77.8 | Low |
| Mafu MM, et al. (9) | 2022 | Retrospective cross-sectional | Analytical cross-sectional | 7/8 | 87.5 | Low |
| Areba AS, et al. (10) | 2022 | Retrospective cross-sectional | Analytical cross-sectional | 7/8 | 87.5 | Low |
| Holt L, et al. (11) | 2021 | Retrospective cross-sectional | Analytical cross-sectional | 7/8 | 87.5 | Low |
| Kabore FA, et al. (12) | 2021 | Cross-sectional | Prevalence studies | 7/9 | 77.8 | Low |
| Sharma E, et al. (13) | 2021 | Retrospective cross-sectional | Prevalence studies | 7/9 | 77.8 | Low |
| Sereke D, et al. (14) | 2020 | Retrospective cross-sectional | Prevalence studies | 8/9 | 88.9 | Low |
| Derso et al. (15) | 2020 | Retrospective cross-sectional | Analytical cross-sectional | 7/8 | 87.5 | Low |
| Benski AC, et al. (16) | 2020 | Retrospective cross-sectional | Analytical cross-sectional | 8/8 | 100 | Low |
| Yismaw et al. (17) | 2019 | Retrospective cross-sectional | Analytical cross-sectional | 6/8 | 75 | Low |
| Okunola TO, et al. (18) | 2018 | Retrospective cross-sectional | Analytical cross-sectional | 6/8 | 75 | Low |
| Mwangi HR, et al. (19) | 2018 | Case-control | Case-control | 6/10 | 60 | Moderate |
| Bernard L, et al. (20) | 2019 | Retrospective cross-sectional | Analytical cross-sectional | 7/8 | 87.5 | Low |
| Aynie AA, et al. (21) | 2019 | Cross-sectional | Analytical cross-sectional | 8/8 | 100 | Low |
| Ojewola, et al. (22) | 2018 | Retrospective cross-sectional | Analytical cross-sectional | 5/8 | 62.5 | Moderate |
| McCurdie FK, et al. (23) | 2018 | Retrospective cross-sectional | Prevalence studies | 7/8 | 87.5 | Low |
| Ali W, et al. (24) | 2018 | randomized control trial | randomized control trial | 6/10 | 60 | Moderate |
| Mubikayi L, et al. (25) | 2017 | Prospective cohort | Analytical cross-sectional | 7/8 | 87.5 | Low |
| Ouedraogo I, et al. (26) | 2017 | Retrospective cohort | Cohort studies | 11/11 | 100 | Low |
| Hussen S, et al. (27) | 2017 | Retrospective cross-sectional | Prevalence studies | 7/9 | 77.9 | Low |
| Heller A. et al. (28) | 2017 | Prospective follow-up | Prevalence studies | 6/8 | 75 | Low |
| Sori DA, et al. (29) | 2016 | Cross-sectional | Prevalence studies | 9/9 | 100 | Low |
| Priyadarshi V, et al. (30) | 2016 | Retrospective cross-sectional | Prevalence studies | 5/8 | 62.5 | Moderate |
| Loposso M, et al. (31) | 2016 | Retrospective cross-sectional | Analytical cross-sectional | 7/8 | 87.5 | Low |
| Delamou A, et al. (32) | 2016 | Retrospective cohort | Analytical cross-sectional | 8/8 | 100 | Low |
| Paluku JL, et al. (33) | 2015 | Prospective follow-up | Analytical cross-sectional | 5/8 | 62.5 | Moderate |
| Egziabher TG, et al. (34) | 2015 | Retrospective cross-sectional | Analytical cross-sectional | 8/8 | 100 | Low |
| Delamou A, et al. (35) | 2015 | Retrospective cohort | Analytical cross-sectional | 5/8 | 62.5 | Moderate |
| Browning A, et al. (36) | 2015 | Retrospective cross-sectional | Analytical cross-sectional | 5/8 | 62.5 | Moderate |
| Tebeu PM, et al. (37) | 2014 | Retrospective cohort | Cohort studies | 7/11 | 63.6 | Moderate |
| Ahmed Z, et al. (38) | 2013 | Retrospective cross-sectional | Prevalence studies | 8/9 | 88.9 | Low |
| Tebeu PM, et al. (39) | 2013 | Cross-sectional | Analytical cross-sectional | 4/7 | 57.1 | Moderate |
| Hawkins L, et al. (40) | 2013 | Retrospective cross-sectional | Analytical cross-sectional | 5/7 | 71.4 | Low |
| Siddle K, et al. (41) | 2013 | Retrospective cross-sectional | Prevalence studies | 7/9 | 77.8 | Low |
| Tayler-Smith et al. (42) | 2013 | Retrospective cross-sectional | Prevalence studies | 8/9 | 88.9 | Low |
| Barone M, et al. (43) | 2012 | Prospective follow-up | Analytical cross-sectional | 8/8 | 100 | Low |
| Gupta NP, et al. (44) | 2012 | Cross-sectional | Prevalence studies | 6/9 | 66.7 | Moderate |
| Frajzyngier V, et al. (45) | 2012 | Prospective follow-up | Analytical cross-sectional | 8/8 | 100 | Low |
| Abdullah A, et al. (46) | 2012 | Retrospective cross-sectional | Analytical cross-sectional | 8/8 | 100 | Low |
| Singh V, et al. (47) | 2011 | Retrospective cross-sectional | Prevalence studies | 7/9 | 77.8 | Low |
| Kayondo M, et al. (48) | 2011 | Prospective follow-up | Analytical cross-sectional | 6/8 | 75 | Low |
| Sjoveian S, et al. (49) | 2011 | Retrospective cross-sectional | Analytical cross-sectional | 7/8 | 87.5 | Low |
| Munoz O, et al. (50) | 2011 | Cross-sectional | Analytical cross-sectional | 7/8 | 87.5 | Low |
| McFadden E, et al. (51) | 2011 | Retrospective cross-sectional | Analytical cross-sectional | 4/8 | 50 | Moderate |
| Mathur R, et al. (52) | 2010 | Prospective follow-up | Prevalence studies | 6/9 | 66.7 | Low |
| Sachdev PS, et al. (53) | 2009 | Cross-sectional | Survey | 7/9 | 77.8 | Low |
| Nielsen HS, (54) | 2009 | Prospective follow-up | Prevalence studies | 7/8 | 87.5 | Low |
| Nardos R, et al. (55) | 2009 | Retrospective cross-sectional | Analytical cross-sectional | 7/8 | 87.5 | Low |
| Singh S, et al. (56) | 2009 | Retrospective cross-sectional | Prevalence studies | 7/9 | 77.8 | Low |
| Ezzat M, et al. (57) | 2009 | Cross-sectional | Prevalence studies | 4/8 | 50 | Moderate |
| Raassen TJIP, et al. (58) | 2008 | Prospective follow-up | Prevalence studies | 8/9 | 88.9 | Low |
| Shafqat T, et al. (59) | 2009 | Cross-sectional | Prevalence studies | 4/8 | 50 | Moderate |
| Goh JTW, et al. (60) | 2008 | Prospective follow-up | Analytical cross-sectional | 8/8 | 100 | Low |
| Browning A, et al. (61) | 2008 | Prospective follow-up | Prevalence studies | 9/9 | 100 | Low |
| Uprety DK, et al (62) | 2008 | Retrospective cross-sectional | Prevalence studies | 6/9 | 66.7 | Moderate |
| Nafiou I, et al. (63) | 2007 | Cross-sectional | Prevalence studies | 7/9 | 77.8 | Low |
| Holme A, et al. (64) | 2007 | Retrospective cross-sectional | Analytical cross-sectional | 7/8 | 87.5 | Low |
| Al-Beiti MAM and Lu X. (65) | 2007 | Retrospective cross-sectional | Prevalence studies | 6/9 | 66.7 | Moderate |
| Roenneburg ML, et al. (66) | 2006 | Prospective follow-up | Prevalence studies | 5/8 | 62.5 | Moderate |
| Husain A, et al. (67) | 2005 | Cross-sectional | Prevalence studies | 5/8 | 62.5 | Moderate |
| Ahmad S, et al. (68) | 2005 | Retrospective cross-sectional | Prevalence studies | 6/9 | 66.7 | Moderate |
| Naru T, et al. (69) | 2004 | Prospective follow-up | Prevalence studies | 5/8 | 62.5 | Moderate |
| Rafique M. et al. (70) | 2002 | Cross-sectional | Prevalence studies | 4/8 | 50 | Moderate |
| Rangnekar NP, et al. (71) | 2000 | Retrospective cohort | Cohort studies | 8/11 | 72.7 | Low |
| Kelly J, et al. (72) | 1998 | Cross-sectional | Prevalence studies | 7/9 | 77.8 | Low |
| Hilton P, et al. (73) | 1998 | Retrospective cross-sectional | Prevalence studies | 5/9 | 55.6 | Moderate |
| Elkins TE. (74) | 1994 | Prospective follow-up | Prevalence studies | 5/9 | 55.6 | Moderate |
| Kelly J. et al. (75) | 1993 | Retrospective cross-sectional | Prevalence studies | 9/9 | 100 | Low |
| Raut V, et al. (76) | 1993 | Retrospective cross-sectional | Prevalence studies | 6/8 | 75 | Low |
| Wadhawan S, et al. (77) | 1983 | Retrospective cross-sectional | Prevalence studies | 8/9 | 88.9 | Low |
| Kelly J. (78) | 1979 | Cross-sectional | Prevalence studies | 7/9 | 77.8 | Low |
| Rao KB. (79) | 1971 | Cross-sectional | Prevalence studies | 5/9 | 55.6 | Moderate |

1. Gezimu W, Sime T, Diriba A, Gemechu D. Repair failure and associated factors among women who underwent obstetric fistula surgery in Southwest Ethiopia: A retrospective study. Women's Health. 2023;19:17455057231192325.

2. Traore TM, Ouedraogo S, Kabore M, Traore JJ. Characteristics of obstetric urogenital fistulas in a regional teaching hospital in Burkina Faso: a retrospective cross-sectional study. The Pan African medical journal. 2023;44:105.

3. Kumsa Meikena H, Bihon AM, Serka S. Predictors and outcomes of surgical repair of obstetric fistula at Mekelle Hamlin Fistula Center, Northern Ethiopia. International urogynecology journal. 2023.

4. Niragira J, Wang TX. Treatment of Vesico-Vaginal Fistula by General Practitioners trained in Fistula Repair owing to a lack of surgeons in rural areas in Burundi. Tropical doctor. 2023;53(2):207-9.

5. Patel TB, Nisarata HR, Patel VK, Wagh MR. Obstetric Fistula Surgery: Indicators of Recurrence and Successful Treatment. Research Journal of Medical Sciences. 2023;17(4):445-9.

6. Ambese TY, Gebre H, Berhe A, Fisseha G, Gufue ZH, Hailu NA, et al. Effect of vaginal scarring on the recovery of surgical repair of obstetric fistula in Northern Ethiopia. Int J Gynecol Obstet. 2023;160(3):915-25.

7. Tadesse S, Ejigu N, Edosa D, Ashegu T, Dulla D. Obstetric fistula repair failure and its associated factors among women underwent repair in Yirgalem Hamlin fistula center, Sidama Regional State, Southern Ethiopia, 2021: a retrospective cross sectional study. BMC women's health. 2022;22(1):288.

8. Asif M, Abrar S, Abrar T. Causative factors, social and surgical outcomes of vesicovaginal fistulas treated via an abdominal transvesical approach: A single-center experience. International Journal of Gynecology and Obstetrics. 2023;160(1):209-13.

9. Mafu MM, Banze DFK, Aussak BTT, Kolié D, Camara BS, Nembunzu D, et al. Factors associated with surgical repair success of female genital fistula in the Democratic Republic of Congo: Experiences of the Fistula Care Plus Project, 2017–2019. Trop Med Int Health. 2022;27(9):831-9.

10. Areba AS, Akessa GM, Tadesse M, Haile A, Abire BG, Eritero AC, et al. Recovery Time and Its Predictors among Women Admitted with Obstetric Fistula in Jimma University Medical Center Southwest, Ethiopia: A Retrospective Cohort Study. medRxiv. 2022.

11. Holt L, Potluri T, Tanner JP, Duffy S, Wasingya L, Greene K. Risk factors for early and late failures following repair of urogenital fistulas. International urogynecology journal. 2021;32(9):2473-82.

12. Kabore FA, Nama SDA, Ouedraogo B, Kabore M, Ouattara A, Kirakoya B, et al. Characteristics of Obstetric and Iatrogenic Urogenital Fistulas in Burkina Faso: A Cross-Sectional Study. Advances in Urology. 2021;2021((Kabore, Nama, Kabore, Kirakoya) Department of Urology and Andrology, University Hospital Yalgado Ouedraogo of Ouagadougou, Ouagadougou, Burkina Faso(Ouedraogo) Urology Department, University Hospital of Tingandogo, Tingandogo, Burkina Faso(Ouattara) Urol):8838146.

13. Sharma E, Iqbal M, Masood S. Five-Year Retrospective Study on the Management of Vesicovaginal Fistula from a Tertiary Care Centre of Jammu. JK Science. 2021;23(4):191-4.

14. Sereke D, Hailemelecot H, Issak Y, Estifanose D. Obstetric Vesico-vaginal Fistulae: A Documentary Review of Women Managed in Mendefera Zonal Referral and National Fistula Hospital, Eritrea. Science. 2020;8(5):149-54.

15. Derso EA, Ayalew S, Eshete A, Wale M. Determinants of time to recovery from obstetric fistula by using the data of university of Gondar teaching hospital fistula center, Gondar -Ethiopia: A parametric survival regression analysis. Cogent Medicine. 2020;7(1):1816259.

16. Benski AC, Delavy M, Rochat CH, Viviano M, Catarino R, Elsig V, et al. Prognostic factors and long-term outcomes of obstetric fistula care using the Tanguiéta model. Int J Gynecol Obstet. 2020;148(3):331-7.

17. Yismaw L, Alemu K, Addis A, Alene M. Time to recovery from obstetric fistula and determinants in Gondar university teaching and referral hospital, northwest Ethiopia. BMC women's health. 2019;19(1):5.

18. Okunola TO, Yakubu E, Daniyan B, Ekwedigwe K, Eliboh M, Sunday-Adeoye I. Profile and outcome of patients with recurrent urogenital fistula in a fistula centre in Nigeria. International urogynecology journal. 2019;30(2):197-201.

19. Mwangi HR, Wang'ombe A, Mabeya H, Kipruto H, Wanjala A. Factors associated with obstetric fistula repair failure among women admitted at Gynocare Women’s and Fistula Hospital in Kenya, 2012-2016: a case control study. Nepal Journal of Obstetrics and Gynaecology. 2018;13(2).

20. Bernard L, Giles A, Fabiano S, Giles S, Hudgins S, Olson A, et al. Predictors of Obstetric Fistula Repair Outcomes in Lubango, Angola. Journal of Obstetrics and Gynaecology Canada. 2019;41(12):1726-33.

21. Aynie A, Yihunie A, Mekonnen A. Magnitude of repair failure and associated factors among women undergone obstetric fistula repair in Bahir Dar Hamlin Fistula Center, Amhara Region, Northwest Ethiopia. International Journal of Scientific Reports. 2019;5:324.

22. Ojewola RW, Tijani KH, Jeje EA, Ogunjimi MA, Animashaun EA, Akanmu ON. Transabdominal repair of vesicovaginal fistulae: A 10-year tertiary care hospital experience in Nigeria. The Nigerian postgraduate medical journal. 2018;25(4):213-9.

23. McCurdie FK, Moffatt J, Jones K. Vesicovaginal fistula in Uganda. Journal of Obstetrics and Gynaecology. 2018;38(6):822-7.

24. Ali W, Kharal IA, Ijaz I, Younis M. Outcome of transvaginal vesicovaginal fistula repair with martius fat pad flap in comparison to simple closure. Pak J Med Health Sci. 2018;12(3):1126-8.

25. Mubikayi L, Matson DO, Lokomba V, Mboloko J, Kamba JP, Tozin R. Determinants of Outcomes and Prognosis Score in Obstetric Vesico-Vaginal Fistula Repair. Open Journal of Obstetrics and Gynecology. 2017;Vol.07No.07:11.

26. Ouedraogo I, Payne C, Nardos R, Adelman AJ, Wall LL. Obstetric fistula in Niger: 6-month postoperative follow-up of 384 patients from the Danja Fistula Center. International urogynecology journal. 2018;29(3):345-51.

27. Hussen S, Melese E. Time-to-recovery from obstetric fistula and associated factors: The case of Harar Hamlin Fistula Center. Ethiop J Health Dev. 2017;31(2):85-95.

28. Heller A. Demographic profile and treatment outcomes of 100 women with obstetric fistula in Niger. Proceedings in Obstetrics and Gynecology. 2017;7:1-15.

29. Sori DA, Azale AW, Gemeda DH. Characteristics and repair outcome of patients with Vesicovaginal fistula managed in Jimma University teaching Hospital, Ethiopia. BMC Urology. 2016;16(1):41.

30. Priyadarshi V, Singh JP, Bera MK, Kundu AK, Pal DK. Genitourinary Fistula: An Indian Perspective. J Obstet Gynaecol India. 2016;66(3):180-4.

31. Loposso M, Hakim L, Ndundu J, Lufuma S, Punga A, De Ridder D. Predictors of Recurrence and Successful Treatment Following Obstetric Fistula Surgery. Urology. 2016;97:80-5.

32. Delamou A, Delvaux T, Beavogui AH, Toure A, Kolié D, Sidibé S, et al. Factors associated with the failure of obstetric fistula repair in Guinea: implications for practice. Reproductive Health. 2016;13(1):135.

33. Paluku JL, Carter TE. Obstetric vesico-vaginal fistulae seen in the Northern Democratic Republic of Congo: a descriptive study. Afr Health Sci. 2015;15(4):1104-11.

34. Egziabher TG, Eugene N, Ben K, Fredrick K. Obstetric fistula management and predictors of successful closure among women attending a public tertiary hospital in Rwanda: a retrospective review of records. BMC research notes. 2015;8:774.

35. Delamou A, Diallo M, Beavogui AH, Delvaux T, Millimono S, Kourouma M, et al. Good clinical outcomes from a 7-year holistic programme of fistula repair in Guinea. Tropical Medicine and International Health. 2015;20(6):813-9.

36. Browning A, Whiteside S. Characteristics, management, and outcomes of repair of rectovaginal fistula among 1100 consecutive cases of female genital tract fistula in Ethiopia. International journal of gynaecology and obstetrics: the official organ of the International Federation of Gynaecology and Obstetrics. 2015;131(1):70-3.

37. Tebeu PM, Maninzou SD, Takam D, Nguefack-Tsague G, Fomulu JN, Rochat CH. Surgical outcome following treatment of obstetric vesicovaginal fistula among HIV-positive and HIV-negative patients in Cameroon. International Journal of Gynecology and Obstetrics. 2014;125(2):168-9.

38. Ahmed Z, Abdullahi H, Yola A, Yakasai I. Obstetrics fistula repairs in Kano, Northern Nigeria: The journey so far. Annals of Tropical Medicine and Public Health. 2013;6(5):545-8.

39. Tebeu PM, Fosso GK, Vadandi V, Dohbit JS, Fomulu JN, Rochat CH. Prognostic value of repeated surgery on obstetric vesico-vaginal fistula outcome: A Cameroonian experience. Asian Pacific Journal of Reproduction. 2013;2(4):330-2.

40. Hawkins L, Spitzer RF, Christoffersen-Deb A, Leah J, Mabeya H. Characteristics and surgical success of patients presenting for repair of obstetric fistula in western Kenya. Int J Gynecol Obstet. 2013;120(2):178-82.

41. Siddle K, Vieren L, Fiander A. Characterising women with obstetric fistula and urogenital tract injuries in Tanzania. International urogynecology journal. 2014;25(2):249-55.

42. Tayler-Smith K, Zachariah R, Manzi M, van den Boogaard W, Vandeborne A, Bishinga A, et al. Obstetric Fistula in Burundi: a comprehensive approach to managing women with this neglected disease. BMC pregnancy and childbirth. 2013;13(1):164.

43. Barone M, Frajzyngier V, Ruminjo J, Asiimwe F, Hamidou Barry T, Bello A, et al. Determinants of fistula repair post-operative outcomes: A prospective cohort study. International Journal of Gynecology and Obstetrics. 2012;119(SUPPL. 3):S167.

44. Gupta NP, Mishra S, Mishra A, Seth A, Anand A. Outcome of repeat supratrigonal obstetric vesicovaginal fistula repair after previous failed repair. Urologia internationalis. 2012;88(3):259-62.

45. Frajzyngier V, Ruminjo J, Asiimwe F, Barry TH, Bello A, Danladi D, et al. Factors influencing choice of surgical route of repair of genitourinary fistula, and the influence of route of repair on surgical outcomes: findings from a prospective cohort study. BJOG Int J Obstet Gynaecol. 2012;119(11):1344-53.

46. Abdullah A, Javed A, Syed S, Farooqui N. Doctor, will i be dry? factors determining recurrence after vesicovaginal fistula repair. Urology. 2012;80(3 SUPPL. 1):S146-S7.

47. Singh V, Sinha RJ, Mehrotra S, Sankhwar SN, Bhatt S. Repair of vesicovaginal fistula by the transabdominal route: outcome at a north Indian tertiary hospital. International urogynecology journal. 2012;23(4):411-6.

48. Kayondo M, Wasswa S, Kabakyenga J, Mukiibi N, Senkungu J, Stenson A, et al. Predictors and outcome of surgical repair of obstetric fistula at a regional referral hospital, Mbarara, western Uganda. BMC Urology. 2011;11(1):23-.

49. Sjoveian S, Vangen S, Mukwege D, Onsrud M. Surgical outcome of obstetric fistula: A retrospective analysis of 595 patients. Acta obstetricia et gynecologica Scandinavica. 2011;90(7):753-60.

50. Munoz O, Bowling CB, Gerten KA, Taryor R, Norman AM, Szychowski JM, et al. Factors influencing post-operative short-term outcomes of vesicovaginal fistula repairs in a community hospital in Liberia. British Journal of Medical and Surgical Urology. 2011;4(6):259-65.

51. McFadden E, Taleski SJ, Bocking A, Spitzer RF, Mabeya H. Retrospective review of predisposing factors and surgical outcomes in obstetric fistula patients at a single teaching hospital in Western Kenya. Journal of obstetrics and gynaecology Canada : JOGC = Journal d'obstetrique et gynecologie du Canada : JOGC. 2011;33(1):30-5.

52. Mathur R, Joshi N, Aggarwal G, Raikwar R, Shrivastava V, Mathur P, et al. Urogenital fistulae: A prospective study of 50 cases at a tertiary care hospital. Urology annals. 2010;2(2):67-70.

53. Sachdev PS, Hassan N, Abbasi RM, Das CM. Genito-urinary fistula: a major morbidity in developing countries. Journal of Ayub Medical College, Abbottabad : JAMC. 2009;21(2):8-11.

54. Nielsen HS, Lindberg L, Nygaard U, Aytenfisu H, Johnston OL, Sørensen B, et al. A community-based long-term follow up of women undergoing obstetric fistula repair in rural Ethiopia. BJOG Int J Obstet Gynaecol. 2009;116(9):1258-64.

55. Nardos R, Browning A, Chen CCG. Risk factors that predict failure after vaginal repair of obstetric vesicovaginal fistulae. American journal of obstetrics and gynecology. 2009;200(5):578.e1-.e4.

56. Singh S, Chandhiok N, Singh Dhillon B. Obstetric fistula in India: current scenario. International urogynecology journal and pelvic floor dysfunction. 2009;20(12):1403-5.

57. Ezzat M, Ezzat MM, Tran VQ, Aboseif SR. Repair of giant vesicovaginal fistulas. J Urol. 2009;181(3):1184-8.

58. Raassen TJIP, Verdaasdonk EGG, Vierhout ME. Prospective results after first-time surgery for obstetric fistulas in East African women. International urogynecology journal. 2008;19(1):73-9.

59. Shafqat T, Faiz NR, Haleemi M. PROFILE AND REPAIR SUCCESS OF VESICOVAGINAL FISTULA IN NWFP. Journal of Postgraduate Medical Institute. 2011;23(1).

60. Goh JTW, Browning A, Berhan B, Chang A. Predicting the risk of failure of closure of obstetric fistula and residual urinary incontinence using a classification system. International urogynecology journal. 2008;19(12):1659-62.

61. Browning A, Menber B. Women with obstetric fistula in Ethiopia: A 6-month follow up after surgical treatment. BJOG: An International Journal of Obstetrics and Gynaecology. 2008;115(12):1564-9.

62. Uprety DK, Subedi S, Budhathoki B, Regmi MC. Vesicovaginal fistula at tertiary care center in eastern Nepal. Journal of the Nepal Medical Association. 2008;47(171):120-2.

63. Nafiou I, Idrissa A, Ghaïchatou AK, Roenneburg ML, Wheeless CR, Genadry RR. Obstetric vesico-vaginal fistulas at the National Hospital of Niamey, Niger. International journal of gynaecology and obstetrics: the official organ of the International Federation of Gynaecology and Obstetrics. 2007;99 Suppl 1:S71-4.

64. Holme A, Breen M, MacArthur C. Obstetric fistulae: A study of women managed at the Monze Mission Hospital, Zambia. BJOG: An International Journal of Obstetrics and Gynaecology. 2007;114(8):1010-7.

65. Al-Beiti MAM, Lu X. Genital tract fistulae in the Republic of Yemen, Sana'a. Journal of Medical Sciences. 2007;7(3):473-9.

66. Roenneburg ML, Genadry R, Wheeless CR, Jr. Repair of obstetric vesicovaginal fistulas in Africa. American journal of obstetrics and gynecology. 2006;195(6):1748-52.

67. Husain A, Johnson K, Glowacki CA, Osias J, Wheeless Jr CR, Asrat K, et al. Surgical management of complex obstetric fistula in Eritrea. Journal of Women's Health. 2005;14(9):839-44.

68. Ahmad S, Nishtar A, Hafeez GA, Khan Z. Management of vesico-vaginal fistulas in women. International journal of gynaecology and obstetrics: the official organ of the International Federation of Gynaecology and Obstetrics. 2005;88(1):71-5.

69. Naru T, Rizvi JH, Talati J. Surgical repair of genital fistulae. Journal of Obstetrics and Gynaecology Research. 2004;30(4):293-6.

70. Rafique M. Genitourinary fistulas of obstetric origin. Int Urol Nephrol. 2002;34(4):489-93.

71. Rangnekar NP, Imdad Ali N, Kaul SA, Pathak HR. Role of the martins procedure in the management of urinary-vaginal fistulas. J Am Coll Surg. 2000;191(3):259-63.

72. Kelly J. Vesico-vaginal and recto-vaginal fistulae. J Obstet Gynaecol. 1998;18(3):249-51.

73. Hilton P, Ward A. Epidemiological and surgical aspects of urogenital fistulae: A review of 25 years' experience in southeast nigeria. International urogynecology journal. 1998;9(4):189-94.

74. Elkins TE. Surgery for the obstetric vesicovaginal fistula: a review of 100 operations in 82 patients. American journal of obstetrics and gynecology. 1994;170(4):1108-18; discussion 18.

75. Kelly J, Kwast B. Epidemiologic study of vesicovaginal fistulas in Ethiopia. International urogynecology journal. 1993;4:278-81.

76. Raut V, Bhattacharya M. Vesical fistulae--an experience from a developing country. J Postgrad Med. 1993;39(1):20-1.

77. Wadhawan S, Wacha DS. A review of urinary fistulae in a university teaching hospital. International journal of gynaecology and obstetrics: the official organ of the International Federation of Gynaecology and Obstetrics. 1983;21(5):381-5.

78. Kelly J. Vesicovaginal Fistulae. Br J Urol. 1979;51(3):208-10.

79. Rao KBMDDGO. Urinary Fistulae of Obstetric Origin. J Obstet Gynaecol Res. 1971;2(2):81-3.
