# Supplementary material for: Proportions and determinants of successful surgical repair of obstetric fistula in low- and middle-income countries: A systematic review and meta-analysis
Source: PLoS One. 2024 May 9;19(5):e0303020. doi: 10.1371/journal.pone.0303020 (PMC11081269; doi:10.1371/journal.pone.0303020)
Supplement: S2 Table — (DOCX) [file pone.0303020.s004.docx]

| **Author** | **Publication Year** | **Country** | **Region** | **Design** | **Sampling method** | **Sample size** | **Data collection method** | **Data retrieval period** | **Type of fistula** | **Route of surgery** | **Assessment period** |
| --- | --- | --- | --- | --- | --- | --- | --- | --- | --- | --- | --- |
| Gezimu W, et al. (1) | 2023 | Ethiopia | Africa | Retrospective cross-sectional | Simple random sampling | 385 | Card review | 2015 to 2020 | Not specified | Not specified | At 3 weeks |
| Traore TM, et al. (2) | 2023 | Burkina Faso | Africa | Retrospective cross-sectional | Survey | 50 | Card review | 2015 to 2019 | VVF and RVF | Vaginal, abdominal, and combined | at 3 months |
| Kumsa MH, et al. (3) | 2023 | Ethiopia | Africa | Retrospective cross-sectional | Simple random sampling | 328 | Card review | January 2015 to January 2020 | VVF and RVF | vaginal and abdominal | Immediately |
| Niragira J, Wang TX (4) | 2023 | Burundi | Africa | Cross-sectional | Survey | 70 | Card review | June 2020 to May 2021 | VVF | Vaginal, abdominal and combined | at 3 months |
| Patel TB, et al (5) | 2023 | India | South-East Asia | Retrospective cross-sectional | Survey | 75 | Card review | Not Specified | Multiple | Vaginal | at 3 months |
| Ambese TY, et al. (6) | 2022 | Ethiopia | Africa | Retrospective cohort | Simple random sampling | 224 | Card review | January 2015 to February 2020 | VVF and RVF | Not specified | At 3 weeks |
| Tadesse et al. (7) | 2022 | Ethiopia | Africa | Cross-sectional | Survey | 562 | Card review | 2016 to 2020 | VVF and RVF | Abdominal | Not specified |
| Asif M, et al (8) | 2022 | Pakistan | Eastern Mediterranean | Cross-sectional | Survey | 120 | Card review | 2014 to 2019 | VVF | Abdominal | At discharge |
| Mafu MM, et al. (9) | 2022 | DRC | Africa | Retrospective cross-sectional | Survey | 895 | Card review | 2017 to 2019 | VVF and RVF | Vaginal, abdominal, and combined | At discharge |
| Areba AS, et al. (10) | 2022 | Ethiopia | Africa | Retrospective cross-sectional | Survey | 270 | Card review | 2011 to 2017 | VVF and RVF | vaginal and abdominal | At discharge |
| Holt L, et al. (11) | 2021 | Uganda | Africa | Retrospective cross-sectional | Survey | 541 | Card review | 2013 to 2019 | VVF and RVF | Vaginal, abdominal, and combined | At discharge |
| Kabore FA, et al. (12) | 2021 | Burkina Faso | Africa | Cross-sectional | Survey | 230 | Interview and examination | 2010 to 2016 | Multiple | Not specified | at 3 months |
| Sharma E, et al. (13) | 2021 | India | South-East Asia | Retrospective cross-sectional | Survey | 39 | Interview | 2015 to 2020 | VVF | vaginal and abdominal | Not specified |
| Sereke D, et al. (14) | 2020 | Eritrea | Africa | Retrospective cross-sectional | Survey | 133 | Card review | 2014 to 2018 | VVF | Not specified | Not specified |
| Derso et al. (15) | 2020 | Ethiopia | Africa | Retrospective cross-sectional | Survey | 289 | Card review | 2014 to 2015 | Not specified | Not specified | Not specified |
| Benski AC, et al. (16) | 2020 | Burkina Faso | Africa | Retrospective cross-sectional | Survey | 274 | Interview | 2008 to 2016 | Not specified | Vaginal, abdominal, and combined | At 12 months |
| Yismaw et al. (17) | 2019 | Ethiopia | Africa | Retrospective cross-sectional | Simple random sampling | 612 | Card review | 2010 to 2017 | Not specified | Not specified | At discharge |
| Okunola TO, et al. (18) | 2018 | Nigeria | Africa | Retrospective cross-sectional | Survey | 154 | Card review | 2014 to 2016 | Not specified | Not specified | at 3 months |
| Mwangi HR, et al. (19) | 2018 | Kenya | Africa | Case-control | Simple random sampling | 357 | Card review | 2012 to 2016 | VVF | Vaginal and Abdominal | At 2 weeks |
| Bernard L, et al. (20) | 2019 | Angola | Africa | Retrospective cross-sectional | Survey | 332 | Card review | 2011 to 2016 | VVF | Not specified | Not specified |
| Aynie AA, et al. (21) | 2019 | Ethiopia | Africa | Cross-sectional | Systematic random sampling | 385 | Card review | 2013 to 2017 | VVF and RVF | Vaginal and abdominal | At discharge |
| Ojewola, et al. (22) | 2018 | Nigeria | Africa | Retrospective cross-sectional | Survey | 53 | Card review | 2008 to 2017 | VVF | Abdominal | Immediately |
| McCurdie FK, et al. (23) | 2018 | Uganda | Africa | Retrospective cross-sectional | Survey | 93 | Interview | July 2013 to July 2014 | VVF | Not specified | At discharge |
| Ali W, et al. (24) | 2018 | Pakistan | Eastern Mediterranean | randomized control trial | Survey | 120 | Interview and examination | Jan 2016 to June 2016 | VVF | Not specified | At 7 days |
| Mubikayi L, et al. (25) | 2017 | DRC | Africa | Prospective cohort | Survey | 483 | Interview | 2011 t0 2014 | VVF and RVF | Not specified | At discharge |
| Ouedraogo I, et al. (26) | 2017 | Niger | Africa | Retrospective cohort | Survey | 134 | Card review | January 2013 to July 2014 | Multiple | Not specified | At discharge |
| Hussen S, et al. (27) | 2017 | Ethiopia | Africa | Retrospective cross-sectional | Survey | 433 | Card review | 2008 to 2014 | Not specified | Not specified | At discharge |
| Heller A. et al. (28) | 2017 | Niger | Africa | Prospective follow-up | Survey | 61 | Interview | 2011 to 2014 | Not specified | Not specified | At discharge |
| Sori DA, et al. (29) | 2016 | Ethiopia | Africa | Cross-sectional | Survey | 168 | Card review | 2011 to 2014 | VVF and RVF | Vaginal and abdominal | At discharge |
| Priyadarshi V, et al. (30) | 2016 | India | South-East Asia | Retrospective cross-sectional | Survey | 41 | Card review | 2007 to 2013 | Multiple | Vaginal and abdominal | Not specified |
| Loposso M, et al. (31) | 2016 | DRC | Africa | Retrospective cross-sectional | Survey | 166 | Card review | 2007 to 2013 | VVF and RVF | Vaginal | at 3 months |
| Delamou A, et al. (32) | 2016 | Guinea | Africa | Retrospective cohort | Survey | 754 | Card review | January 2012 to October 2013 | VVF and RVF | Vaginal, abdominal, and combined | At discharge |
| Paluku JL, et al. (33) | 2015 | DRC | Africa | Prospective follow-up | Survey | 163 | Interview | August 2012 to October 2013 | VVF | Not specified | Immediately |
| Egziabher TG, et al. (34) | 2015 | Rwanda | Africa | Retrospective cross-sectional | Survey | 272 | Card review | 2007 to 2013 | Multiple | vaginal and abdominal | At discharge |
| Delamou A, et al. (35) | 2015 | Guinea | Africa | Retrospective cohort | Survey | 2116 | Card review | January 2007 to September 2013 | VVF and RVF | Not specified | At discharge |
| Browning A, et al. (36) | 2015 | Ethiopia | Africa | Retrospective cross-sectional | Survey | 993 | Card review | January 2005 to October 2008 | VVF and RVF | Vaginal | At discharge |
| Tebeu PM, et al. (37) | 2014 | Cameroon | Africa | Retrospective cohort | Survey | 52 | Card review | January 2009 to March 2012 | VVF | Not specified | At discharge |
| Ahmed Z, et al. (38) | 2013 | Nigeria | Africa | Retrospective cross-sectional | Survey | 262 | Card review | October 2011 to September 2012 | VVF and RVF | Vaginal | At discharge |
| Tebeu PM, et al. (39) | 2013 | Cameroon | Africa | Cross-sectional | Survey | 81 | Card review | 2005 to 2011 | VVF | vaginal and abdominal | At discharge |
| Hawkins L, et al. (40) | 2013 | Kenya | Africa | Retrospective cross-sectional | Survey | 260 | Card review | January 2005 to July 2010 | VVF and RVF | Not specified | At 2 weeks |
| Siddle K, et al. (41) | 2013 | Tanzania | Africa | Retrospective cross-sectional | Survey | 217 | Card review | 2012 | VVF and RVF | Not specified | At discharge |
| Tayler-Smith et al. (42) | 2013 | Burundi | Africa | Retrospective cross-sectional | Survey | 454 | Card review | July 2010 to December 2011 | VVF and RVF | Not specified | At discharge |
| Barone M, et al. (43) | 2012 | Multiple (Bangladesh, Guinea, Niger, Nigeria and Uganda) | South-East Asia and Africa | Prospective follow-up | Survey | 1274 | Interview | September 2007 to September 2010 | Multiple | Not specified | at 3 months |
| Gupta NP, et al. (44) | 2012 | India | South-East Asia | Cross-sectional | Survey | 68 | Interview and examination | 2002 to 2007 | VVF | Abdominal | Immediately |
| Frajzyngier V, et al. (45) | 2012 | (Uganda, Guinea, Niger, Nigeria, Bangladesh) | South-East Asia and Africa | Prospective follow-up | Survey | 1274 |  | September 2007 to September 2010 | Multiple | Vaginal and abdominal | At 3 months |
| Abdullah A, et al. (46) | 2012 | Pakistan | Eastern Mediterranean | Retrospective cross-sectional | Survey | 640 | Card review | January 2007 to June 2012 | VVF | Vaginal and abdominal | Immediately |
| Singh V, et al. (47) | 2011 | India | South-East Asia | Retrospective cross-sectional | Survey | 48 | Card review | 2001 to 2010 | VVF | Abdominal | At 3 weeks |
| Kayondo M, et al. (48) | 2011 | Uganda | Africa | Prospective follow-up | Survey | 69 | Card review | February 2010 to June 2010 | VVF and RVF | Not specified | At discharge |
| Sjoveian S, et al. (49) | 2011 | DRC | Africa | Retrospective cross-sectional | Survey | 665 | Card review | November 2005 to November 2007 | VVF | Vaginal | At discharge |
| Munoz O, et al. (50) | 2011 | Liberia | Africa | Cross-sectional | Survey | 40 | Card review and examination | June 2008 to January 2009 | VVF | vaginal and abdominal | At 2 weeks |
| McFadden E, et al. (51) | 2011 | Kenya | Africa | Retrospective cross-sectional | Survey | 82 | Card review | 1999 to 2007 | VVF and RVF | Vaginal, abdominal, and combined | At discharge |
| Mathur R, et al. (52) | 2010 | India | South-East Asia | Prospective follow-up | Survey | 50 | Interview and examination | July 2005 to July 2009 | Multiple | Vaginal, abdominal, and combined | At discharge |
| Sachdev PS, et al. (53) | 2009 | Pakistan | Eastern Mediterranean | Cross-sectional | Survey | 268 | Interview and examination | June 1996 to December 2007 | VVF | Vaginal and abdominal | Not specified |
| Nielsen HS, (54) | 2009 | Ethiopia | Africa | Prospective follow-up | Survey | 44 | Interview and examination | December 2004 to July 2006 | Not specified | Vaginal | At discharge |
| Nardos R, et al. (55) | 2009 | Ethiopia | Africa | Retrospective cross-sectional | Survey | 1045 | Card review | 2006 to 2007 | VVF | Not specified | Immediately |
| Singh S, et al. (56) | 2009 | India | South-East Asia | Retrospective cross-sectional | Survey | 322 | Card review | 2000 to 2006 | VVF and RVF | Not specified | Not specified |
| Ezzat M, et al. (57) | 2009 | Egypt | Eastern Mediterranean | Cross-sectional | Survey | 35 | Card review | 1980 to 2007 | VVF | Vaginal and abdominal | Immediately |
| Raassen TJIP, et al. (58) | 2008 | Multiple (Kenya, Tanzania and Uganda) | Africa | Prospective follow-up | Survey | 565 | Interview and examination | January 2001 to August 2003 | VVF | Vaginal and abdominal | At 2 weeks |
| Shafqat T, et al. (59) | 2009 | Pakistan | Eastern Mediterranean | Cross-sectional | Survey | 38 | Interview and examination | Jan to Dec 2006 | VVF | Vaginal | At discharge |
| Goh JTW, et al. (60) | 2008 | Ethiopia | Africa | Prospective follow-up | Survey | 987 | Interview and examination | September 2004 to February 2007 | VVF | Vaginal | At discharge |
| Browning A, et al. (61) | 2008 | Ethiopia | Africa | Prospective follow-up | Survey | 390 | Interview | Not Specified | VVF and RVF | Vaginal | At discharge |
| Uprety DK, et al (62) | 2008 | Nepal | South-East Asia | Retrospective cross-sectional | Survey | 23 | Card review | 2005 to 2007 | VVF | Vaginal and abdominal | At discharge |
| Nafiou I, et al. (63) | 2007 | Niger | Africa | Cross-sectional | Survey | 104 | Interview and examination | December 2003 to February 2005 | VVF | Vaginal, abdominal and combined | At 3 months |
| Holme A, et al. (64) | 2007 | Zambia | Africa | Retrospective cross-sectional | Survey | 252 | Card review | August 2003 to December 2005 | VVF and RVF | Not specified | Immediately |
| Al-Beiti MAM and Lu X. (65) | 2007 | Yemen | Eastern Mediterranean | Retrospective cross-sectional | Survey | 51 | Card review | 1997 to 2004 | Multiple | Not specified | Not specified |
| Roenneburg ML, et al. (66) | 2006 | Niger | Africa | Prospective follow-up | Survey | 65 | Interview and examination | October 2003 to April 2005 | VVF | Vaginal, abdominal, and combined | Immediately |
| Husain A, et al. (67) | 2005 | Eritrea | Africa | Cross-sectional | Survey | 31 | Interview | Sep-04 | Urethro vaginal fistula | vaginal and abdominal | 4 weeks |
| Ahmad S, et al. (68) | 2005 | Pakistan | Eastern Mediterranean | Retrospective cross-sectional | Survey | 1086 | Card review | Not Specified | Multiple | Vaginal and abdominal | Immediately |
| Naru T, et al. (69) | 2004 | Pakistan | Eastern Mediterranean | Prospective follow-up | Survey | 68 | Interview and examination | 1988 to 2002 | Multiple | vaginal and abdominal | At 8 weeks |
| Rafique M. et al. (70) | 2002 | Pakistan | Eastern Mediterranean | Cross-sectional | Survey | 42 | Interview and examination | December 1999 to May 2002 | Multiple | Vaginal and abdominal | at 3 months |
| Rangnekar NP, et al. (71) | 2000 | India | South-East Asia | Retrospective cohort | Survey | 46 | Card review | January 1994 to January 1999 | VVF | Vaginal | At 3 weeks |
| Kelly J, et al. (72) | 1998 | Ethiopia | Africa | Cross-sectional | Survey | 716 | Card review | Not Specified | VVF and RVF | vaginal and abdominal | Immediately |
| Hilton P, et al. (73) | 1998 | Nigeria | Africa | Retrospective cross-sectional | Survey | 2360 | Card review | 1970 to 1994 | Multiple | Vaginal and abdominal | Immediately |
| Elkins TE. (74) | 1994 | Multiple (Ghana and Nigeria) | Africa | Prospective follow-up | Survey | 82 | Card review | 1975 to 1993 | Multiple | vaginal and abdominal | Immediately |
| Kelly J. et al. (75) | 1993 | Ethiopia | Africa | Retrospective cross-sectional | Systematic random sampling | 309 | Card review | 1983 to 1988 | Multiple | vaginal and abdominal | Immediately |
| Raut V, et al. (76) | 1993 | India | South-East Asia | Retrospective cross-sectional | Survey | 62 | Card review | 1981 to 1990 | Multiple | Vaginal and abdominal | At discharge |
| Wadhawan S, et al. (77) | 1983 | Zambia | Africa | Retrospective cross-sectional | Survey | 44 | Card review | 1974 to 1981 | Multiple | vaginal and abdominal | Immediately |
| Kelly J. (78) | 1979 | Ethiopia | Africa | Cross-sectional | Survey | 128 | Interview and examination | Not Specified | VVF and RVF | Vaginal and abdominal | At 3 weeks |
| Rao KB. (79) | 1971 | India | South-East Asia | Cross-sectional | Survey | 260(1, 4) | Card review | Not Specified | Multiple | Vaginal | Immediately |

1. Gezimu W, Sime T, Diriba A, Gemechu D. Repair failure and associated factors among women who underwent obstetric fistula surgery in Southwest Ethiopia: A retrospective study. Women's Health. 2023;19:17455057231192325.

2. Traore TM, Ouedraogo S, Kabore M, Traore JJ. Characteristics of obstetric urogenital fistulas in a regional teaching hospital in Burkina Faso: a retrospective cross-sectional study. The Pan African medical journal. 2023;44:105.

3. Kumsa Meikena H, Bihon AM, Serka S. Predictors and outcomes of surgical repair of obstetric fistula at Mekelle Hamlin Fistula Center, Northern Ethiopia. International urogynecology journal. 2023.

4. Niragira J, Wang TX. Treatment of Vesico-Vaginal Fistula by General Practitioners trained in Fistula Repair owing to a lack of surgeons in rural areas in Burundi. Tropical doctor. 2023;53(2):207-9.

5. Patel TB, Nisarata HR, Patel VK, Wagh MR. Obstetric Fistula Surgery: Indicators of Recurrence and Successful Treatment. Research Journal of Medical Sciences. 2023;17(4):445-9.

6. Ambese TY, Gebre H, Berhe A, Fisseha G, Gufue ZH, Hailu NA, et al. Effect of vaginal scarring on the recovery of surgical repair of obstetric fistula in Northern Ethiopia. Int J Gynecol Obstet. 2023;160(3):915-25.

7. Tadesse S, Ejigu N, Edosa D, Ashegu T, Dulla D. Obstetric fistula repair failure and its associated factors among women underwent repair in Yirgalem Hamlin fistula center, Sidama Regional State, Southern Ethiopia, 2021: a retrospective cross sectional study. BMC women's health. 2022;22(1):288.

8. Asif M, Abrar S, Abrar T. Causative factors, social and surgical outcomes of vesicovaginal fistulas treated via an abdominal transvesical approach: A single-center experience. International Journal of Gynecology and Obstetrics. 2023;160(1):209-13.

9. Mafu MM, Banze DFK, Aussak BTT, Kolié D, Camara BS, Nembunzu D, et al. Factors associated with surgical repair success of female genital fistula in the Democratic Republic of Congo: Experiences of the Fistula Care Plus Project, 2017–2019. Trop Med Int Health. 2022;27(9):831-9.

10. Areba AS, Akessa GM, Tadesse M, Haile A, Abire BG, Eritero AC, et al. Recovery Time and Its Predictors among Women Admitted with Obstetric Fistula in Jimma University Medical Center Southwest, Ethiopia: A Retrospective Cohort Study. medRxiv. 2022.

11. Holt L, Potluri T, Tanner JP, Duffy S, Wasingya L, Greene K. Risk factors for early and late failures following repair of urogenital fistulas. International urogynecology journal. 2021;32(9):2473-82.

12. Kabore FA, Nama SDA, Ouedraogo B, Kabore M, Ouattara A, Kirakoya B, et al. Characteristics of Obstetric and Iatrogenic Urogenital Fistulas in Burkina Faso: A Cross-Sectional Study. Advances in Urology. 2021;2021((Kabore, Nama, Kabore, Kirakoya) Department of Urology and Andrology, University Hospital Yalgado Ouedraogo of Ouagadougou, Ouagadougou, Burkina Faso(Ouedraogo) Urology Department, University Hospital of Tingandogo, Tingandogo, Burkina Faso(Ouattara) Urol):8838146.

13. Sharma E, Iqbal M, Masood S. Five-Year Retrospective Study on the Management of Vesicovaginal Fistula from a Tertiary Care Centre of Jammu. JK Science. 2021;23(4):191-4.

14. Sereke D, Hailemelecot H, Issak Y, Estifanose D. Obstetric Vesico-vaginal Fistulae: A Documentary Review of Women Managed in Mendefera Zonal Referral and National Fistula Hospital, Eritrea. Science. 2020;8(5):149-54.

15. Derso EA, Ayalew S, Eshete A, Wale M. Determinants of time to recovery from obstetric fistula by using the data of university of Gondar teaching hospital fistula center, Gondar -Ethiopia: A parametric survival regression analysis. Cogent Medicine. 2020;7(1):1816259.

16. Benski AC, Delavy M, Rochat CH, Viviano M, Catarino R, Elsig V, et al. Prognostic factors and long-term outcomes of obstetric fistula care using the Tanguiéta model. Int J Gynecol Obstet. 2020;148(3):331-7.

17. Yismaw L, Alemu K, Addis A, Alene M. Time to recovery from obstetric fistula and determinants in Gondar university teaching and referral hospital, northwest Ethiopia. BMC women's health. 2019;19(1):5.

18. Okunola TO, Yakubu E, Daniyan B, Ekwedigwe K, Eliboh M, Sunday-Adeoye I. Profile and outcome of patients with recurrent urogenital fistula in a fistula centre in Nigeria. International urogynecology journal. 2019;30(2):197-201.

19. Mwangi HR, Wang'ombe A, Mabeya H, Kipruto H, Wanjala A. Factors associated with obstetric fistula repair failure among women admitted at Gynocare Women’s and Fistula Hospital in Kenya, 2012-2016: a case control study. Nepal Journal of Obstetrics and Gynaecology. 2018;13(2).

20. Bernard L, Giles A, Fabiano S, Giles S, Hudgins S, Olson A, et al. Predictors of Obstetric Fistula Repair Outcomes in Lubango, Angola. Journal of Obstetrics and Gynaecology Canada. 2019;41(12):1726-33.

21. Aynie A, Yihunie A, Mekonnen A. Magnitude of repair failure and associated factors among women undergone obstetric fistula repair in Bahir Dar Hamlin Fistula Center, Amhara Region, Northwest Ethiopia. International Journal of Scientific Reports. 2019;5:324.

22. Ojewola RW, Tijani KH, Jeje EA, Ogunjimi MA, Animashaun EA, Akanmu ON. Transabdominal repair of vesicovaginal fistulae: A 10-year tertiary care hospital experience in Nigeria. The Nigerian postgraduate medical journal. 2018;25(4):213-9.

23. McCurdie FK, Moffatt J, Jones K. Vesicovaginal fistula in Uganda. Journal of Obstetrics and Gynaecology. 2018;38(6):822-7.

24. Ali W, Kharal IA, Ijaz I, Younis M. Outcome of transvaginal vesicovaginal fistula repair with martius fat pad flap in comparison to simple closure. Pak J Med Health Sci. 2018;12(3):1126-8.

25. Mubikayi L, Matson DO, Lokomba V, Mboloko J, Kamba JP, Tozin R. Determinants of Outcomes and Prognosis Score in Obstetric Vesico-Vaginal Fistula Repair. Open Journal of Obstetrics and Gynecology. 2017;Vol.07No.07:11.

26. Ouedraogo I, Payne C, Nardos R, Adelman AJ, Wall LL. Obstetric fistula in Niger: 6-month postoperative follow-up of 384 patients from the Danja Fistula Center. International urogynecology journal. 2018;29(3):345-51.

27. Hussen S, Melese E. Time-to-recovery from obstetric fistula and associated factors: The case of Harar Hamlin Fistula Center. Ethiop J Health Dev. 2017;31(2):85-95.

28. Heller A. Demographic profile and treatment outcomes of 100 women with obstetric fistula in Niger. Proceedings in Obstetrics and Gynecology. 2017;7:1-15.

29. Sori DA, Azale AW, Gemeda DH. Characteristics and repair outcome of patients with Vesicovaginal fistula managed in Jimma University teaching Hospital, Ethiopia. BMC Urology. 2016;16(1):41.

30. Priyadarshi V, Singh JP, Bera MK, Kundu AK, Pal DK. Genitourinary Fistula: An Indian Perspective. J Obstet Gynaecol India. 2016;66(3):180-4.

31. Loposso M, Hakim L, Ndundu J, Lufuma S, Punga A, De Ridder D. Predictors of Recurrence and Successful Treatment Following Obstetric Fistula Surgery. Urology. 2016;97:80-5.

32. Delamou A, Delvaux T, Beavogui AH, Toure A, Kolié D, Sidibé S, et al. Factors associated with the failure of obstetric fistula repair in Guinea: implications for practice. Reproductive Health. 2016;13(1):135.

33. Paluku JL, Carter TE. Obstetric vesico-vaginal fistulae seen in the Northern Democratic Republic of Congo: a descriptive study. Afr Health Sci. 2015;15(4):1104-11.

34. Egziabher TG, Eugene N, Ben K, Fredrick K. Obstetric fistula management and predictors of successful closure among women attending a public tertiary hospital in Rwanda: a retrospective review of records. BMC research notes. 2015;8:774.

35. Delamou A, Diallo M, Beavogui AH, Delvaux T, Millimono S, Kourouma M, et al. Good clinical outcomes from a 7-year holistic programme of fistula repair in Guinea. Tropical Medicine and International Health. 2015;20(6):813-9.

36. Browning A, Whiteside S. Characteristics, management, and outcomes of repair of rectovaginal fistula among 1100 consecutive cases of female genital tract fistula in Ethiopia. International journal of gynaecology and obstetrics: the official organ of the International Federation of Gynaecology and Obstetrics. 2015;131(1):70-3.

37. Tebeu PM, Maninzou SD, Takam D, Nguefack-Tsague G, Fomulu JN, Rochat CH. Surgical outcome following treatment of obstetric vesicovaginal fistula among HIV-positive and HIV-negative patients in Cameroon. International Journal of Gynecology and Obstetrics. 2014;125(2):168-9.

38. Ahmed Z, Abdullahi H, Yola A, Yakasai I. Obstetrics fistula repairs in Kano, Northern Nigeria: The journey so far. Annals of Tropical Medicine and Public Health. 2013;6(5):545-8.

39. Tebeu PM, Fosso GK, Vadandi V, Dohbit JS, Fomulu JN, Rochat CH. Prognostic value of repeated surgery on obstetric vesico-vaginal fistula outcome: A Cameroonian experience. Asian Pacific Journal of Reproduction. 2013;2(4):330-2.

40. Hawkins L, Spitzer RF, Christoffersen-Deb A, Leah J, Mabeya H. Characteristics and surgical success of patients presenting for repair of obstetric fistula in western Kenya. Int J Gynecol Obstet. 2013;120(2):178-82.

41. Siddle K, Vieren L, Fiander A. Characterising women with obstetric fistula and urogenital tract injuries in Tanzania. International urogynecology journal. 2014;25(2):249-55.

42. Tayler-Smith K, Zachariah R, Manzi M, van den Boogaard W, Vandeborne A, Bishinga A, et al. Obstetric Fistula in Burundi: a comprehensive approach to managing women with this neglected disease. BMC pregnancy and childbirth. 2013;13(1):164.

43. Barone M, Frajzyngier V, Ruminjo J, Asiimwe F, Hamidou Barry T, Bello A, et al. Determinants of fistula repair post-operative outcomes: A prospective cohort study. International Journal of Gynecology and Obstetrics. 2012;119(SUPPL. 3):S167.

44. Gupta NP, Mishra S, Mishra A, Seth A, Anand A. Outcome of repeat supratrigonal obstetric vesicovaginal fistula repair after previous failed repair. Urologia internationalis. 2012;88(3):259-62.

45. Frajzyngier V, Ruminjo J, Asiimwe F, Barry TH, Bello A, Danladi D, et al. Factors influencing choice of surgical route of repair of genitourinary fistula, and the influence of route of repair on surgical outcomes: findings from a prospective cohort study. BJOG Int J Obstet Gynaecol. 2012;119(11):1344-53.

46. Abdullah A, Javed A, Syed S, Farooqui N. Doctor, will i be dry? factors determining recurrence after vesicovaginal fistula repair. Urology. 2012;80(3 SUPPL. 1):S146-S7.

47. Singh V, Sinha RJ, Mehrotra S, Sankhwar SN, Bhatt S. Repair of vesicovaginal fistula by the transabdominal route: outcome at a north Indian tertiary hospital. International urogynecology journal. 2012;23(4):411-6.

48. Kayondo M, Wasswa S, Kabakyenga J, Mukiibi N, Senkungu J, Stenson A, et al. Predictors and outcome of surgical repair of obstetric fistula at a regional referral hospital, Mbarara, western Uganda. BMC Urology. 2011;11(1):23-.

49. Sjoveian S, Vangen S, Mukwege D, Onsrud M. Surgical outcome of obstetric fistula: A retrospective analysis of 595 patients. Acta obstetricia et gynecologica Scandinavica. 2011;90(7):753-60.

50. Munoz O, Bowling CB, Gerten KA, Taryor R, Norman AM, Szychowski JM, et al. Factors influencing post-operative short-term outcomes of vesicovaginal fistula repairs in a community hospital in Liberia. British Journal of Medical and Surgical Urology. 2011;4(6):259-65.

51. McFadden E, Taleski SJ, Bocking A, Spitzer RF, Mabeya H. Retrospective review of predisposing factors and surgical outcomes in obstetric fistula patients at a single teaching hospital in Western Kenya. Journal of obstetrics and gynaecology Canada : JOGC = Journal d'obstetrique et gynecologie du Canada : JOGC. 2011;33(1):30-5.

52. Mathur R, Joshi N, Aggarwal G, Raikwar R, Shrivastava V, Mathur P, et al. Urogenital fistulae: A prospective study of 50 cases at a tertiary care hospital. Urology annals. 2010;2(2):67-70.

53. Sachdev PS, Hassan N, Abbasi RM, Das CM. Genito-urinary fistula: a major morbidity in developing countries. Journal of Ayub Medical College, Abbottabad : JAMC. 2009;21(2):8-11.

54. Nielsen HS, Lindberg L, Nygaard U, Aytenfisu H, Johnston OL, Sørensen B, et al. A community-based long-term follow up of women undergoing obstetric fistula repair in rural Ethiopia. BJOG Int J Obstet Gynaecol. 2009;116(9):1258-64.

55. Nardos R, Browning A, Chen CCG. Risk factors that predict failure after vaginal repair of obstetric vesicovaginal fistulae. American journal of obstetrics and gynecology. 2009;200(5):578.e1-.e4.

56. Singh S, Chandhiok N, Singh Dhillon B. Obstetric fistula in India: current scenario. International urogynecology journal and pelvic floor dysfunction. 2009;20(12):1403-5.

57. Ezzat M, Ezzat MM, Tran VQ, Aboseif SR. Repair of giant vesicovaginal fistulas. J Urol. 2009;181(3):1184-8.

58. Raassen TJIP, Verdaasdonk EGG, Vierhout ME. Prospective results after first-time surgery for obstetric fistulas in East African women. International urogynecology journal. 2008;19(1):73-9.

59. Shafqat T, Faiz NR, Haleemi M. PROFILE AND REPAIR SUCCESS OF VESICOVAGINAL FISTULA IN NWFP. Journal of Postgraduate Medical Institute. 2011;23(1).

60. Goh JTW, Browning A, Berhan B, Chang A. Predicting the risk of failure of closure of obstetric fistula and residual urinary incontinence using a classification system. International urogynecology journal. 2008;19(12):1659-62.

61. Browning A, Menber B. Women with obstetric fistula in Ethiopia: A 6-month follow up after surgical treatment. BJOG: An International Journal of Obstetrics and Gynaecology. 2008;115(12):1564-9.

62. Uprety DK, Subedi S, Budhathoki B, Regmi MC. Vesicovaginal fistula at tertiary care center in eastern Nepal. Journal of the Nepal Medical Association. 2008;47(171):120-2.

63. Nafiou I, Idrissa A, Ghaïchatou AK, Roenneburg ML, Wheeless CR, Genadry RR. Obstetric vesico-vaginal fistulas at the National Hospital of Niamey, Niger. International journal of gynaecology and obstetrics: the official organ of the International Federation of Gynaecology and Obstetrics. 2007;99 Suppl 1:S71-4.

64. Holme A, Breen M, MacArthur C. Obstetric fistulae: A study of women managed at the Monze Mission Hospital, Zambia. BJOG: An International Journal of Obstetrics and Gynaecology. 2007;114(8):1010-7.

65. Al-Beiti MAM, Lu X. Genital tract fistulae in the Republic of Yemen, Sana'a. Journal of Medical Sciences. 2007;7(3):473-9.

66. Roenneburg ML, Genadry R, Wheeless CR, Jr. Repair of obstetric vesicovaginal fistulas in Africa. American journal of obstetrics and gynecology. 2006;195(6):1748-52.

67. Husain A, Johnson K, Glowacki CA, Osias J, Wheeless Jr CR, Asrat K, et al. Surgical management of complex obstetric fistula in Eritrea. Journal of Women's Health. 2005;14(9):839-44.

68. Ahmad S, Nishtar A, Hafeez GA, Khan Z. Management of vesico-vaginal fistulas in women. International journal of gynaecology and obstetrics: the official organ of the International Federation of Gynaecology and Obstetrics. 2005;88(1):71-5.

69. Naru T, Rizvi JH, Talati J. Surgical repair of genital fistulae. Journal of Obstetrics and Gynaecology Research. 2004;30(4):293-6.

70. Rafique M. Genitourinary fistulas of obstetric origin. Int Urol Nephrol. 2002;34(4):489-93.

71. Rangnekar NP, Imdad Ali N, Kaul SA, Pathak HR. Role of the martins procedure in the management of urinary-vaginal fistulas. J Am Coll Surg. 2000;191(3):259-63.

72. Kelly J. Vesico-vaginal and recto-vaginal fistulae. J Obstet Gynaecol. 1998;18(3):249-51.

73. Hilton P, Ward A. Epidemiological and surgical aspects of urogenital fistulae: A review of 25 years' experience in southeast nigeria. International urogynecology journal. 1998;9(4):189-94.

74. Elkins TE. Surgery for the obstetric vesicovaginal fistula: a review of 100 operations in 82 patients. American journal of obstetrics and gynecology. 1994;170(4):1108-18; discussion 18.

75. Kelly J, Kwast B. Epidemiologic study of vesicovaginal fistulas in Ethiopia. International urogynecology journal. 1993;4:278-81.

76. Raut V, Bhattacharya M. Vesical fistulae--an experience from a developing country. J Postgrad Med. 1993;39(1):20-1.

77. Wadhawan S, Wacha DS. A review of urinary fistulae in a university teaching hospital. International journal of gynaecology and obstetrics: the official organ of the International Federation of Gynaecology and Obstetrics. 1983;21(5):381-5.

78. Kelly J. Vesicovaginal Fistulae. Br J Urol. 1979;51(3):208-10.

79. Rao KBMDDGO. Urinary Fistulae of Obstetric Origin. J Obstet Gynaecol Res. 1971;2(2):81-3.
