# Supplementary material for: Proportions and determinants of successful surgical repair of obstetric fistula in low- and middle-income countries: A systematic review and meta-analysis
Source: PLoS One. 2024 May 9;19(5):e0303020. doi: 10.1371/journal.pone.0303020 (PMC11081269; doi:10.1371/journal.pone.0303020)
Supplement: Attachment — Submitted filename: Point by point response.docx [file pone.0303020.s006.docx]

*Dear the Editor and reviewers,*

*We, the authors, are highly grateful for your contribution to the publication process of the manuscript. We have taken a look at the manuscript and made amendments based on your feedback. Please find the point-by-point response described below. Please don’t hesitate to get back to the corresponding author in case you have concerns to be addressed further.*

**Response for the Editor**

1. Please ensure that your manuscript meets PLOS ONE's style requirements, including those for file naming.

Response: We have made the following amendments to match with the PLOS ONE’s publication criteria

a. Headings font size

- 18pt size for heading 1
- 16pt size for heading 2
- 14pt size for heading 3

b. citation, caption and format adjustments for figures, tables, and supplementary files as per the journal’s guidelines.

2. In the online submission form, you indicated that data supporting this study are available from the corresponding author upon reasonable request.

All PLOS journals now require all data underlying the findings described in their manuscript to be freely available to other researchers, either 1. In a public repository, 2. Within the manuscript itself, or 3. Uploaded as supplementary information.

Response: The supporting dataset was uploaded as a supplementary file

3. Please include captions for your Supporting Information files at the end of your manuscript, and update any in-text citations to match accordingly. Please see our Supporting Information guidelines for more information: http://journals.plos.org/plosone/s/supporting-information.

Response: All the supporting files are captioned at the end of the manuscript

4. Please review your reference list to ensure that it is complete and correct. If you have cited papers that have been retracted, please include the rationale for doing so in the manuscript text, or remove these references and replace them with relevant current references. Any changes to the reference list should be mentioned in the rebuttal letter that accompanies your revised manuscript. If you need to cite a retracted article, indicate the article’s retracted status in the References list and also include a citation and full reference for the retraction notice.

Response: We have revised the references, mainly citing the references in square brackets.

**Response for the Reviewers**

Reviewer 1: No comment

Reviewer 2

1. Authors are recommended to check the publisher guideline and edit the draft adhering to the guideline: in particular formatting, reference management (style, and uniformity) etc. <https://journals.plos.org/plosone/s/submission-guidelines>

Response: We have made amendments in line with the journal's guidelines specifically on the font size, citations and captions of tables and figures, and referencing.

2. I believe this statement may be partly untrue (Introduction section, last paragarph) “However, to the authors' knowledge, no systematic review and meta-analysis study has been conducted on the proportion of successful surgical repair outcomes and determinants of surgical repair outcomes in low- and middle-income countries.”

Response: We have amended the statement as follows “However, very few [25, 26] systematic review and meta-analysis studies were conducted on successful surgical repair outcomes and determinants of obstetric fistula but none of them were covered in low- and middle-income countries comprehensively”.

3. Discussion: It is recommended to have rigorous discussion taking consideration of the systematic reviews and meta-analysis on this topic. There are handful of meta-analysis authors can look for and compare and contrast the findings and have robust discussions. Such meta-analysis can be those including earlier publications or recent data (eg. https://pubmed.ncbi.nlm.nih.gov/34981462/) including data from publications after significant improvements in contemporary practice of fistula repairs (https://link.springer.com/article/10.1007/s11934-017-0708-5 ).

Response: We have made some adjustments in the discussion section based on the reviewer's feedback. However, the articles recommended by the reviewer are not suitable to compare with our study. For instance, in the case of the first article, the predominant cause of the fistula was hysterectomy, a non-obstetric cause. But in our case studies conducted on non-obstetric fistula were excluded. Hence, the outcomes cannot be comparable.
